# Assessment of Breeding Potential of Foxtail Millet Varieties Using a TOPSIS Model Constructed Based on Distinctness, Uniformity, and Stability Test Characteristics

**DOI:** 10.3390/plants13152102

**Published:** 2024-07-29

**Authors:** Jin Yu, Xionghui Bai, Kaixi Zhang, Leyong Feng, Zheng Yu, Xiongfei Jiao, Yaodong Guo

**Affiliations:** 1 Maize Research Institute, Shanxi Agricultural University, Xinzhou 034000, China; xzdusyj7017@163.com (J.Y.); 13935874098@163.com (X.B.); 13453005276@163.com (L.F.); 13935067810@163.com (Z.Y.); 2 Development Center of Science and Technology, MARA, Beijing 100176, China; kaixi0526@163.com

**Keywords:** foxtail millet, DUS, breeding trends, TOPSIS

## Abstract

Foxtail millet (*Setaria italica*) is an important cereal crop with rich nutritional value. Distinctness, Uniformity, and Stability (DUS) are the prerequisites for the application of new variety rights for foxtail millet. In this study, we investigated 32 DUS test characteristics of 183 foxtail millet resources, studied their artificial selection trends, and identified the varieties that conform to breeding trends. The results indicated significant differences in terms of the means, ranges, and coefficients of variation for each characteristic. A correlation analysis was performed to determine the correlations between various DUS characteristics. A principal component analysis was conducted on 31 test characteristics to determine their primary characteristics. By plotting PC1 and PC2, all the germplasm resources could be clearly distinguished. The trends in foxtail millet breeding were identified through a differential analysis of the DUS test characteristics between the landrace and cultivated varieties. Based on these breeding trends, the optimal solution types for multiple evaluation indicators were determined; the weight allocation was calculated; and a specific TOPSIS algorithm was designed to establish a comprehensive multi-criteria decision-making model. Using this model, the breeding potential of foxtail millet germplasm resources were ranked. These findings provided important reference for foxtail millet breeding in the future.

## 1. Introduction

Foxtail millet (*Setaria italica*) is a cereal crop in the Poaceae family, widely distributed in temperate regions where it is used for food or feed purposes [1]. Foxtail millet has been reported to have originated in China [2]. Because of its excellent characteristics such as a short growth cycle, strong adaptability, high yield, and drought resistance, foxtail millet has become a highly favored important cereal crop [3,4,5]. Additionally, compared to other small grain crops, foxtail millet has high nutritional value. It contains unique nutrient components and is gradually becoming a model crop for plant genomic research [6,7,8]. Significant differences are observed in the characteristics of various foxtail millet varieties because of factors such as planting environment and genetic diversity, posing great challenges to foxtail millet breeding. Therefore, it is urgent to accurately understand and explore the breeding trends of foxtail millet.

Before systematic breeding, foxtail millet varieties were local landraces adapted to specific regional growing conditions and gradually evolved through long-term human selection and cultivation from wild green foxtail grass (*Setaria viridis*) [9]. Breeding foxtail millet is closely related to market demand and economic development. Over time, breeding objectives change with variations in the market, and millet varieties cultivated and promoted at different stages exhibit different characteristics. Analyzing and summarizing the trends and characteristics of millet varieties can provide important guidance for future breeding efforts. Numerous reports are available on the evolution of agronomic characteristics in various millet-planting regions. For example, in some studies on millet cultivation in certain regions, researchers have identified a series of millet varieties and observed an increasing trend in yield over time. Key factors such as reduced plant height, early maturity, and improved lodging resistance have played significant roles [10,11,12]. Similar trends were observed for millet varieties in other regions, indicating that millet breeding is a continuous process of optimization and improvement [13,14,15]. These studies provided valuable references for millet breeding and offered the scientific basis for millet cultivation and production.

The Technique for Order Preference by Similarity to Ideal Solution (TOPSIS) algorithm serves as a crucial multi-attribute decision-making tool in assessing the merits of a series of alternative solutions [16]. This algorithm primarily determines the optimal solution based on the relative proximity of the alternative solutions to the ideal and negative ideal solutions. Using the TOPSIS algorithm, various alternative solutions can be quickly and accurately evaluated. Therefore, it is widely applied across various decision-making domains [17,18]. Ali Bagherzadeh et al. used the TOPSIS method to examine the qualitative suitability of different irrigation strategies for wheat crops within the framework of the Food and Agriculture Organization [19]. Similarly, Nayak et al. used the TOPSIS method to identify the most suitable application of rice husk for potential use in energy generation [20]. Furthermore, Enqin Zheng et al. used the TOPSIS algorithm to assess the impact of humic acid on rice yield under various irrigation methods [21]. It is evident that the TOPSIS method holds significant value in evaluating cultivation methods and selecting agricultural intervention measures.

Distinctness, Uniformity, and Stability (DUS) is a process of plant variety identification tests or indoor analytical tests and determines whether a tested variety is a new variety based on the test results of distinctiveness, uniformity, and stability. It provides reliable criteria for the protection of new plant varieties and plays a significant role in plant breeding and new variety protection. Numerous studies have applied DUS testing methods in breeding to ensure the identifiability, distinguishability, and specificity of new varieties; this is performed through comprehensive comparisons and identifications of the morphological and biological characteristics and molecular markers of new varieties, effectively protecting the intellectual property rights of new varieties. For example, Tao Chen et al. improved the statistical phenotypic characteristics for DUS testing through the DUS identification of 143 germplasms and selected excellent germplasm resources for oil tea breeding using the TOPSIS algorithm [22]. Liyuan Wang et al. determined wheat breeding trends by studying the differences in the DUS testing characteristics of wheat in various regions. The application of DUS testing has played a positive role in promoting the sustainable development of breeding strategies [23].

This study comprehensively evaluated 32 phenotypic characteristics of 183 foxtail millet germplasm resources to assess the diversity of and variation in foxtail millet in DUS testing. Specifically, by comparing the performance of landrace and cultivated varieties in these tests, we analyzed the trends in foxtail millet breeding in detail. Ultimately, using the TOPSIS method for establishing a model for ranking, we identified superior varieties aligning with the current breeding trends. These findings provided not only specific guidance and recommendations for foxtail millet cultivation and breeding but also valuable insights to advance research and practical applications in the field of foxtail millet breeding.

## 2. Results

### 2.1. Observation and Analysis of DUS Testing Characteristics

**Figure 1:**
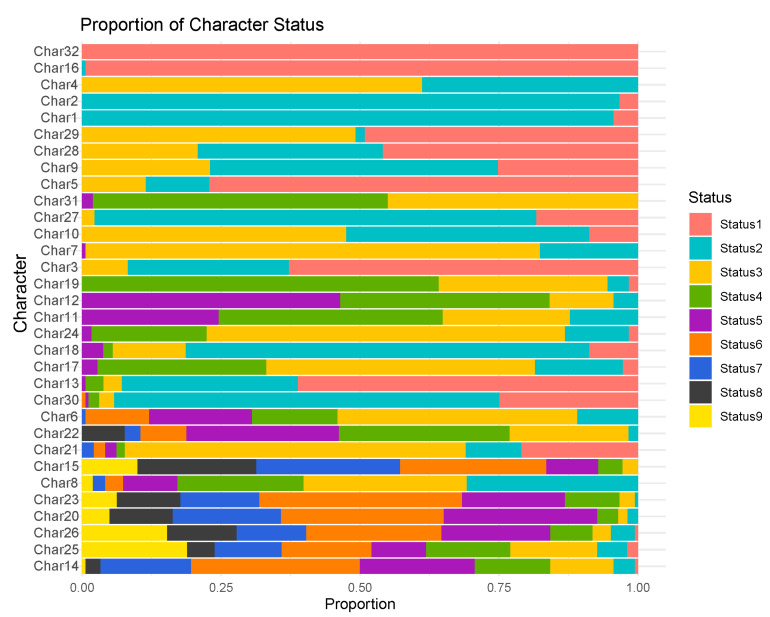
Distribution of variation types of all DUS test characteristics in 183 millet varieties.

The 32 phenotypic characteristics of 183 foxtail millet varieties were observed and analyzed. The results revealed that various phenotypic characteristics exhibited varying frequency distributions (Figure 1). In the foxtail millet germplasm resources, only one type of endosperm (glutinous) was observed. Therefore, this characteristic was not further analyzed. Characteristics 1, 2, 4, and 16 exhibited two expression types. Characteristics 3, 5, and 27 had narrow and single-level distributions. Characteristics 14, 25, and 26 exhibited the widest distribution range, with nine expression levels. Table 1 presents the mean, standard deviation, CV, and Hʹ for the 32 phenotypic characteristics. The CV reflects the dispersion of and variability in data, with a larger coefficient indicating greater variability. Among these characteristics, characteristics 5, 13, and 29 exhibited relatively high variability, with CVs of 50.31%, 50.52%, and 50.13% respectively. Characteristics 1, 2, and 16 exhibited relatively low variability, with CVs of 9.89%, 9.15%, and 3.72% respectively. The characteristics related to yield exhibited extensive variation, with significant differences in grading for the characteristics, such as the panicle length, panicle thickness, number of grains per panicle, and individual panicle weight. Shannon’s diversity index reflects the diversity and evenness of individual distribution. A higher diversity index indicates a more even distribution of individual characteristics in the varieties. Characteristics 14, 15, 20, 22, 23, 25, and 26 had diversity indexes > 1.5. Among them, characteristics 25 and 2 had the highest and lowest diversity indexes of 2.032 and 0.183, respectively.

### 2.2. Correlation of Phenotypic Characteristics

**Figure 2:**
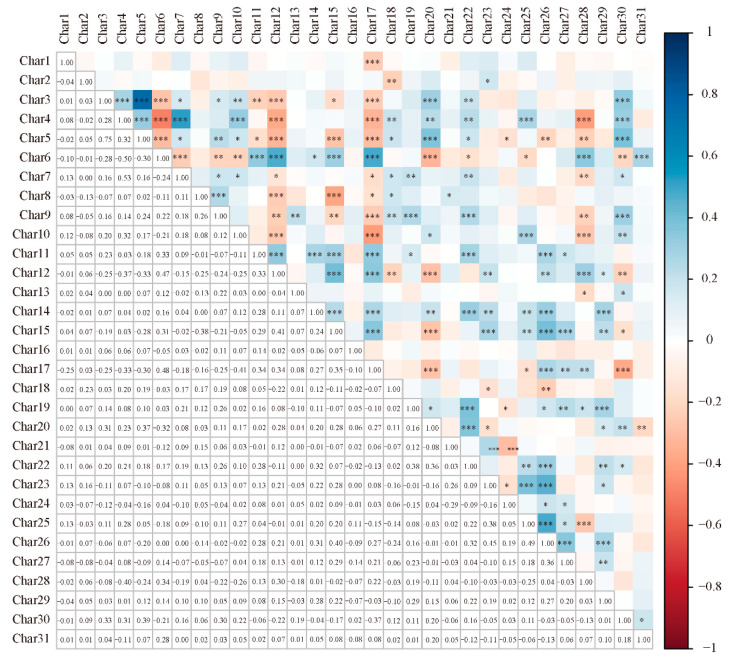
Correlation analysis among DUS testing characteristics of the 183 varieties. *, **, and *** represent significance at *p* < 0.05, 0.01, and 0.001, respectively.

A correlation analysis was conducted on 31 agronomic characteristics (Figure 2). The results revealed various patterns of correlation among the characteristics. The individual panicle weight was significantly correlated with yield-related characteristics, such as the panicle length, panicle thickness, panicle density, and grains per panicle. On an average, each characteristic was correlated with 10.4 other characteristics. Characteristic 16 was not correlated with any other characteristics, whereas character 17 was correlated with the maximum (19) characteristics. Among all the significant correlations, the largest significantly positive correlation (r = 0.75) was observed between characteristics 3 and 5, whereas the largest significantly negative correlation (r = −0.5) was observed between characteristics 4 and 6. Apart from these two correlations, the absolute values of the correlation coefficients between other combinations were ≤0.5, indicating weak correlations.

### 2.3. Cluster Analysis

**Figure 3:**
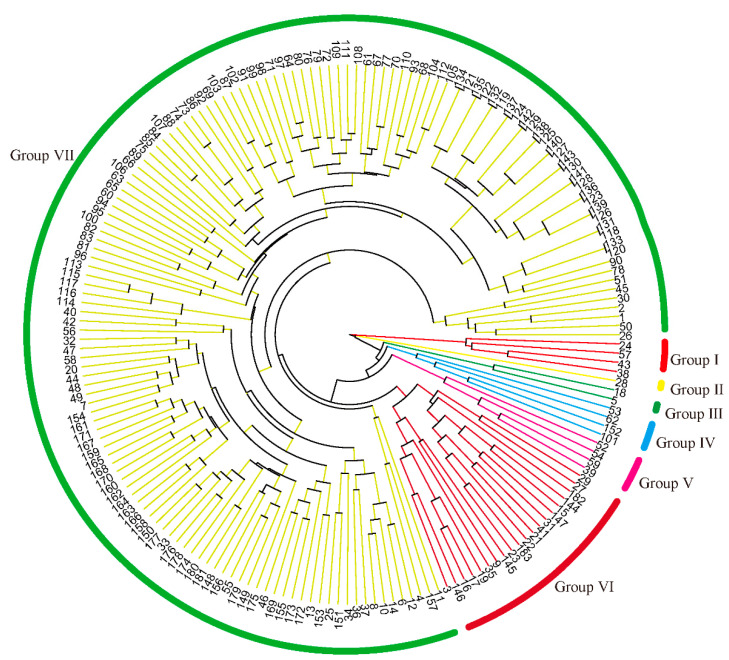
Cluster dendrogram of 183 millet varieties based on DUS testing characteristics.

Based on the data of 32 phenotypic characteristics, the 183 foxtail millet varieties were classified into seven clusters (Figure 3). Cluster 1 consisted of four varieties, Jinfen 111, Datong 29, Qisifeng, and Laohuwei, all exhibiting the highest code values in characteristics 1, 2, 3, 5, 20, 22, 27, 28, and 30. Cluster 2 included only one variety, Huangjinggu, exhibiting the highest code values in characteristics 1, 4, 7, 8, 10, 14, 18, 19, 21, 25, and 27. Clusters 3, 4, and 5 comprised two, four, and four varieties, respectively, each with the highest codes in characteristics 1 and 27. Cluster 6 consisted of 20 varieties with the highest code values in characteristics 1, 6, 9, 11, 12, 13, 17, 24, 29, and 31. Cluster 7 comprised 148 varieties, accounting for 80.9% of the total varieties, exhibiting the highest code values in characteristics 15, 16, 23, and 26.

### 2.4. Principal Component Analysis (PCA)

**Figure 4:**
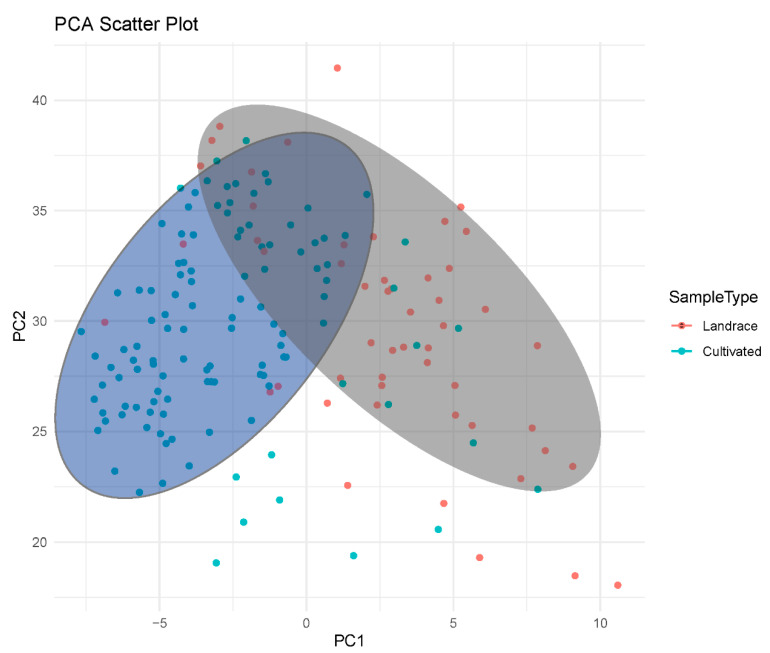
The principal component analysis of the 183 millet varieties. Each point represents a variety, with the blue area indicating the clustering of cultivated varieties and the gray area representing the clustering of landrace varieties.

A PCA was conducted on 183 foxtail millet germplasm resources to identify their major characteristics (Table 2). Overall, 11 significant components were selected, which accounted for 80.79% of the total variance based on eigenvalues > 1. Among them, the first, fifth, and ninth principal components were primarily composed of characteristics related to the seedling stage of foxtail millet (characteristics 1, 2, 3, 4, and 5), referred to as the seedling factor. The second and fourth principal components mainly comprised characteristics related to the panicle of foxtail millet (characteristics 22, 23, 24, 25, and 26), termed as the panicle factor. Characteristics 19 and 28 had a significant loading on the third principal component. The 6th, 7th, and 10th principal components were primarily loaded by individual characteristics (characteristics 16, 17, and 31) as the main negative loading factors. The eighth principal component mainly consisted of characteristics related to the color of the foxtail millet panicles (characteristics 9 and 13), known as the panicle color factor. The 11th principal component was primarily composed of characteristics 16 and 19 but with a lower loading. The projection of all the varieties onto PC1 and PC2 for plotting (Figure 4) demonstrated a clear separation between the landrace and cultivated varieties, indicating significant differences between them.

### 2.5. Analysis of Breeding Trends

**Figure 5:**
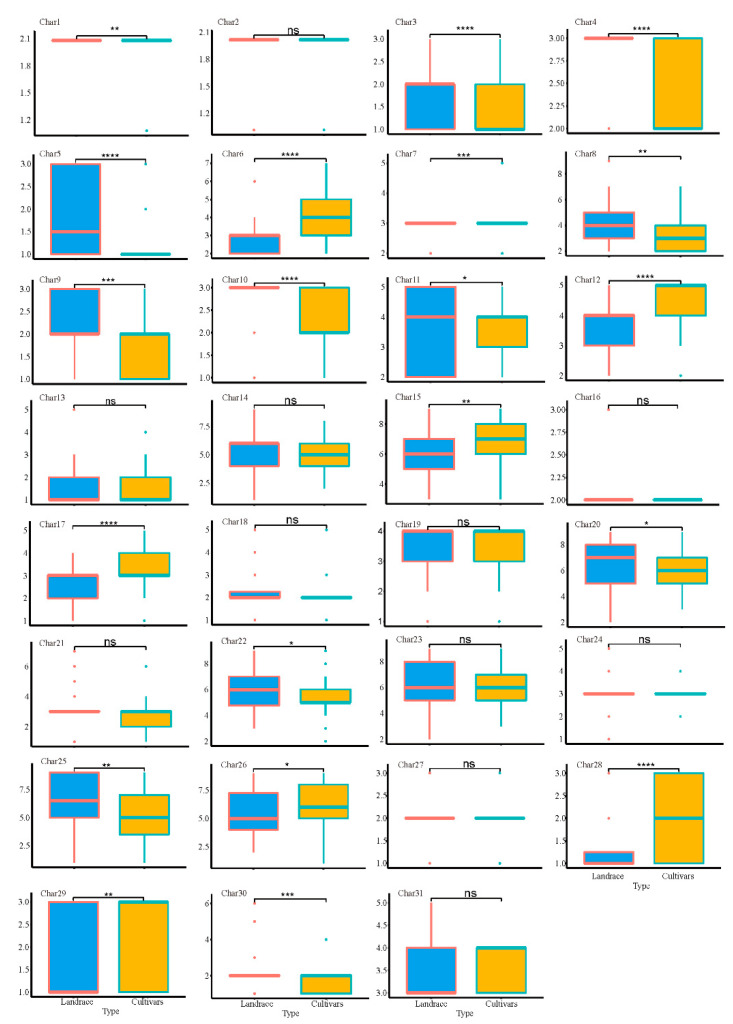
Landrace and cultivated variety differences in all DUS test characters analysis. *, **, *** and **** represent significance at *p* < 0.05, 0.01, 0.001 and 0.0001, respectively. “ns” stands for not significant.

Based on the results, the foxtail millet germplasm resources in this study were divided into two categories: landrace and cultivated varieties (52 and 131 varieties, respectively). A differential analysis of 32 DUS-tested characteristics of foxtail millet (Figure 5) was conducted to predict the current breeding trends in foxtail millet. The differential analysis between the landrace varieties and cultivated varieties revealed that 12 characteristics were not significantly different between the two, indicating that these characteristics are not major factors in the breeding process of foxtail millet. However, significant differences were observed in 20 characteristics between the landrace and cultivated varieties. Specifically, the cultivated varieties exhibited significant superiority over the landraces in characteristics 1 (*p* < 0.01), 6 (*p* < 0.0001), 12 (*p* < 0.01), 15 (*p* < 0.01), 17 (*p* < 0.0001), 26 (*p* < 0.05), 28 (*p* < 0.0001), and 29 (*p* < 0.01). On the other hand, the landraces exhibited significant superiority over the cultivated varieties in characteristics 3 (*p* < 0.0001), 4 (*p* < 0.0001), 5 (*p* < 0.0001), 7 (*p* < 0.001), 8 (*p* < 0.001), 9 (*p* < 0.001), 10 (*p* < 0.0001), 11 (*p* < 0.05), 20 (*p* < 0.05), 22 (*p* < 0.05), 25 (*p* < 0.01), and 30 (*p* < 0.001).

### 2.6. Comprehensive Evaluation Using TOPSIS Algorithm

Based on the identified breeding trends in foxtail millet, the maximum values of the significantly increased characteristics and the minimum values of the significantly decreased characteristics were defined as the positive and negative ideal solutions, respectively. Each characteristic was given equal weight, and a comprehensive multi-criteria decision-making model was established using the TOPSIS algorithm to assess the breeding potential of the seed resources, ranking the landrace and cultivated varieties (Table 3). After computation and analysis, the top 10 varieties were selected in terms of breeding potential. They were Changnong 41, Jinfen 117, Jinxuan 1012, Jinfen 119, Changgu K6, Dayoug 2, Jinfen 110, Jinfen 111, Jingug 21, and Huangjinggu 7.

## 3. Discussion

### 3.1. Phenotypic Variation of Foxtail Millet Resources

The CV is an important indicator for assessing the degree of differences in phenotypic characteristics. It is significantly positively correlated with the degree of phenotypic differences and genetic diversity. This provides greater possibilities for utilizing phenotypic characteristics to identify the varieties and germplasms [23]. The analysis of 32 DUS-tested characteristics of 183 foxtail millet germplasm resources revealed that various characteristics in foxtail millet germplasm resources have a high CV, indicating the presence of rich genetic diversity among foxtail millet germplasm resources. In terms of quantitative characteristics, the median and mean values of the 183 germplasm resources were essentially consistent, reflecting the representativeness of the study subjects. The H′ values of the leaf, stem, and panicle characteristics were relatively high (1.078–2.032), indicating substantial genetic variation in these characteristics. In particular, leaf characteristics reflect the adaptability of plants to various environments and their self-regulation capacity in complex physiological environments; they are considered important indicators for plant science research [24]. In contrast, the H′ values of the grain and seedling characteristics in foxtail millet were lower (0.147–1.045), suggesting that foxtail millet is less affected during the seedling stage and exhibits less characteristic segregation. However, grain characteristics directly impact the yield and quality of foxtail millet and are important characteristics that breeders hope to modify. Nevertheless, due to the low diversity of foxtail millet grains, more constraints are presented for foxtail millet breeding. This emphasizes the importance of correctly identifying breeding trends in foxtail millet breeding.

### 3.2. Correlation Analysis and PCA

A significantly positive correlation was observed between the single panicle weight of foxtail millet and multiple characteristics, including the stem thickness, stem length, length of the second leaf, width of the second leaf, internode number, panicle posture, panicle length, panicle thickness, panicle density, and grain number per panicle. This is consistent with a previous study, indicating that the improvement in foxtail millet yield is related to multiple characteristics [25]. This result is consistent with the source–sink theory [26], where the stem length and thickness of foxtail millet affect the permeability of the nutrients and water in the root system, whereas the increase in the length and width of the second leaf enhances the leaf area and thereby strengthens plant photosynthesis. Additionally, the increase in the panicle length, thickness, density, and grain number per panicle increases the grain yield of foxtail millet. Therefore, the enhancement of foxtail millet yield is influenced by multiple factors. By changing the characteristics related to yield toward the correct breeding trend, the yield of foxtail millet can be improved.

Furthermore, PCA is an effective method for reducing the dimensions of large datasets, enhancing interpretability, reducing information loss, and determining the characteristics that are most suitable and primarily responsible for the variation in the selected materials [27,28]. In this study, the PCA confirmed that the first 11 components explained the majority of the variation, focusing on the characteristics, such as the leaf sheath color in seedlings, leaf posture, leaf hilum anthocyanin coloration, stem length, panicle length, panicle thickness, grain number per panicle, and single panicle weight. These results suggested that these characteristics are suitable for evaluating the genetic diversity of foxtail millet germplasm resources and can be used for phenotypic identification of foxtail millet germplasm resources. Through the analysis, the cultivated and landrace varieties could be clearly divided into two categories, with a certain degree of overlap. This further confirmed the transition from landrace varieties to modern cultivated varieties in the breeding history of foxtail millet. Because the history of foxtail millet breeding is not extensive, a wide range of phenotypic divergence could not be observed between the landrace and cultivated varieties in the breeding process, explaining the presence of the overlap in the PCA.

### 3.3. Analysis of Breeding Trends and Screening of Potential Varietal Resources

Before systematic breeding, foxtail millet varieties were local landraces adapted to specific regional growing conditions, gradually evolved through long-term human selection and cultivation from wild green foxtail grass (*Setaria viridis*) [9]. In the breeding process of foxtail millet, foxtail millet germplasm resources, including landrace varieties and local cultivated varieties, are first collected from various regions and areas [29]. These germplasm resources possess rich genetic variation and adaptability, playing a vital foundational role in foxtail millet breeding [30]. Subsequently, through the evaluation and selection of these landrace varieties, superior individuals or populations with good agronomic and economic characteristics are selected. Further, by employing methods such as controlled hybridization, selection, and progeny screening, the yield, quality, and stress resistance of foxtail millet are gradually enhanced.

Our study determined the breeding trend of foxtail millet by comparing the differences in the DUS test characteristics between the landrace and cultivated varieties. The results indicated significant differences between these in terms of 20 characteristics, with 8 characteristics significantly increasing and 12 characteristics significantly decreasing during the breeding process. Previous studies reported that early cultivated foxtail millet varieties had long awns; however, most modern varieties have short awns [31]. This change is attributed to the vulnerability of early foxtail millet cultivation to damage by birds; long-awned millet varieties are effectively protected against feeding by birds [32]. With advances in modern technology for protection from birds and the decrease in bird populations due to environmental pollution, the length of the awns of foxtail millet gradually shortened. This is consistent with the findings of this study. Grains of cereal plants generally have long and narrow leaves. In this study, the length of the second leaf of foxtail millet gradually decreased, whereas the width of the second leaf increased. Additionally, the plant-to-leaf posture gradually exhibited an upward trend. This can be attributed to changes in the length-to-width ratio of the leaves, allowing them to meet the requirements of modern high-density cultivation, consistent with a previous study [33]. Additionally, the increase in stem thickness enhances the plant’s lodging resistance. Breeders optimize yield by increasing the grain weight of foxtail millet rather than the number of grains per panicle. Reducing the panicle length can make the wheat spikes more compact, reducing the impact of natural factors such as wind or birds on foxtail millet yield and increasing its recoverable rate. The code for the foxtail millet grain shape gradually increases, indicating a transition from ovate to spherical grain shapes. This results in an increase in individual grain volume, further explaining the increase in the thousand-grain weight of foxtail millet.

By constructing a TOPSIS model, this study ranked the breeding potential of foxtail millet germplasm resources, with those ranked higher exhibiting greater breeding potential consistent with the aforementioned breeding trends. Moreover, this model can be used to screen foxtail millet germplasm for subsequent DUS testing by selecting varieties with higher scores. The establishment of this model provided significant guidance for foxtail millet breeding, aiding in the selection of promising foxtail millet germplasm resources for further breeding work and accelerating the foxtail millet breeding process.

## 4. Materials and Methods

### 4.1. Plant Materials and Field Experiments

A total of 183 foxtail millet germplasm resources were collected for this study, comprising 52 landrace varieties and 131 cultivated varieties. The planting experiments were conducted in Xinzhou City, Shanxi Province, China (120° 52′ E, 30° 40′ N; altitude 791 m; annual precipitation 385–516 mm; and average annual temperature 5.0–9.8 °C) in 2022 and 2023. The experiments were designed using a randomized complete block design with three replicates. Each variety was sown in late May, with a minimum of 300 plants per plot planted in 6 rows. Each plot measured 5 m in length and 2.4 m in width. The row spacing was set at 40 cm, and the plant spacing ranged from 7 to 10 cm. The soil at the experimental site was sandy loam. All the experiments were performed according to standard agricultural practices. Organic fertilizer and compound fertilizer were applied at 52,500 and 600 kg·hm^−1^, respectively. After sowing, the experimental plots were irrigated twice: once during the seedling stage and again at the jointing stage using drip irrigation to ensure uniform water distribution. All the irrigation methods were followed as per standard agricultural practices.

### 4.2. Determination of Phenotypic Characteristics and Data Collection

In total, 32 characteristics were investigated as outlined in the foxtail millet DUS testing guidelines (Table 4), comprising 1 qualitative (QL), 14 pseudo-quantitative (PQ), and 17 quantitative (QN) characteristics. The characteristic observation methods included individual visual scoring (VS), population visual scoring (VG), individual measurements (MS), and population measurements (MG). In accordance with the guidelines, the corresponding codes were recorded for the visually scored characteristics. For each measured characteristic (e.g., the leaf length, leaf width, stem length, stem thickness, number of tillers per plant, panicle neck length, panicle length, panicle thickness, number of grains per panicle, individual panicle weight, grain yield, and thousand-grain weight), at least 20 typical plants were selected from each plot for individual measurement and recording.

### 4.3. Statistical Analysis

All the experiments were performed in triplicates. Based on a 2-year investigation and measurements, the mean of each characteristic was used for the statistical analysis. The qualitative and pseudo-qualitative characteristics were classified into 10 grades, 1 grade < X − 2S; 10 grades > X + 2S, with each grade interval being 0.5 s between 1 and 10 grades; X and s are the mean and standard deviation, respectively. The morphological diversity was evaluated using the frequency of characteristic dispersion and Shannon’s diversity index (H′). The minimum value (Min), maximum value (Max), mean, median, standard deviation (SD), coefficient of variation (CV; %), and Hʹ of all the characteristics were measured. The CV for all the quantitative characteristics was calculated as CV¼ S = X, where S is the standard deviation and X is the mean. The H′ for each characteristic was calculated using the following formula: H′ = −Pi ln (Pi) (Pi is the proportion of the individual number of this characteristic in the total individual number). The IBM SPSS Statistics version 20.0 (SPSS Inc., Chicago, IL, USA) was used to estimate the correlation among all the quantitative characteristics with Pearson’s correlation coefficient. A principal component analysis (PCA) was applied to determine the relationship among the individuals. Based on the breeding history of foxtail millet, the foxtail millet varieties were categorized into farmer varieties and breeding lines. The artificial selection trends were determined for the characteristics assessed in the DUS tests of the foxtail millet through a differential analysis between the farmer varieties and breeding lines. The results of the correlation analysis, principal component analysis, and differential analysis were visualized using the R package “ggplot2” (number of the software. 3.3.6). This study employed the TOPSIS method for the multi-attribute decision analysis. Following the differential analysis revealing significant differences among the data, the dataset underwent thorough data cleaning to ensure consistency. In the analysis, all the evaluation criteria were equally weighted. If the newly bred varieties significantly exceeded the local landraces in a specific trait, the ideal solution was set as the maximum value; otherwise, it was set as the minimum value. Using TOPSIS, a model was constructed to evaluate each variety’s comprehensive score based on the proximity score formula, thereby identifying the foxtail millet varieties with the greatest breeding potential. The process was performed using the R package “topsis” (number of the software. 1.0.0).

## 5. Conclusions

In this study, we evaluated the phenotypic diversity and breeding trends of 183 foxtail millet germplasm resources based on 32 phenotypic characteristics. Our results revealed significant variability across multiple traits within the foxtail millet germplasm. Key traits relevant to foxtail millet breeding and germplasm identification were identified through correlation and PCA. Additionally, by analyzing the differential traits of landrace and cultivated varieties in DUS tests, we determined their breeding trends. Based on these trends, we established optimal solution types for multiple evaluation indicators, accordingly allocated weights, and developed a specific TOPSIS algorithm to construct a comprehensive multi-criteria decision-making model. This model was used to rank the breeding potential of foxtail millet germplasm resources. These findings will guide the expansion of the foxtail millet characterization system and optimization of DUS testing guidelines. Furthermore, this study provided a reference for the further utilization of and improvement in major traits in foxtail millet germplasm resources, laying a theoretical foundation for future breeding of new varieties.

## Figures and Tables

**Table 1:** Variability and genetic diversity of all DUS test characters.

Characteristics	Mean	SD	CV	Max	Min	H′
char1	1.96	0.19	9.89	2	1	0.183
char2	1.97	0.18	9.15	2	1	0.147
char3	1.46	0.65	44.08	3	1	0.864
char4	2.63	0.48	18.43	3	2	0.661
char5	1.35	0.68	50.31	3	1	0.707
char6	3.79	1.24	32.70	7	2	1.490
char7	2.83	0.42	14.69	5	2	0.503
char8	3.31	1.31	39.54	9	2	1.493
char9	1.97	0.69	35.21	3	1	1.218
char10	2.40	0.65	26.98	3	1	1.129
char11	3.78	0.96	25.43	5	2	1.489
char12	4.25	0.83	19.55	5	2	1.228
char13	1.51	0.76	50.52	5	1	1.045
char14	5.22	1.51	29.02	9	1	1.790
char15	6.78	1.34	19.79	9	3	1.705
char16	2.01	0.07	3.72	3	2	1.078
char17	3.15	0.82	26.06	5	1	1.431
char18	2.19	0.78	35.39	5	1	1.156
char19	3.56	0.65	18.28	4	1	1.100
char20	6.14	1.36	22.23	9	2	1.709
char21	2.69	1.10	40.59	7	1	1.256
char22	5.37	1.21	22.51	9	2	1.579
char23	6.06	1.44	23.80	9	2	1.737
char24	3.10	0.68	21.84	5	1	1.379
char25	5.66	2.29	40.45	9	1	2.032
char26	6.20	1.91	30.84	9	1	1.949
char27	1.84	0.42	23.11	3	1	0.58
char28	1.74	0.77	44.35	3	1	1.045
char29	1.98	0.99	50.13	3	1	0.766
char30	1.83	0.66	36.25	6	1	0.812
char31	3.55	0.52	14.65	5	3	0.745
char32	2.00	0.00	0.00	2	2	0.000

Note: SD: standard deviation. CV: coefficient of variation. H′: Shannon diversity index.

**Table 2:** The principal component analysis of the 32 quantitative characters in the 183 *Setaria italica* accessions.

Characters	1	2	3	4	5	6	7	8	9	10	11
Char1	0.134	0.097	−0.243	−0.060	−0.114	0.088	−0.286	0.181	0.535	0.110	−0.054
Char2	−0.036	0.124	0.023	−0.143	0.507	−0.140	−0.055	0.271	0.200	−0.372	0.145
Char3	0.579	0.074	0.178	0.096	0.519	−0.001	0.039	−0.209	−0.079	0.207	−0.130
Char4	0.620	0.335	−0.209	0.074	−0.127	0.036	0.214	−0.331	0.116	−0.206	0.140
Char5	0.671	−0.014	0.212	0.123	0.430	0.067	0.176	−0.112	−0.055	0.144	−0.092
Char6	−0.686	−0.021	0.349	0.160	0.054	0.337	−0.040	0.041	0.054	0.104	−0.045
Char7	0.401	0.281	0.007	−0.165	−0.190	0.225	0.190	−0.258	0.372	−0.243	0.209
Char8	0.244	−0.080	0.291	−0.338	−0.570	0.017	−0.093	0.123	−0.243	−0.069	−0.084
Char9	0.456	0.167	0.176	−0.126	−0.178	0.290	0.029	0.458	−0.204	0.184	0.219
Char10	0.449	0.211	−0.189	0.137	−0.129	0.204	−0.220	−0.115	−0.029	−0.003	−0.335
Char11	−0.358	0.429	0.311	0.056	−0.163	0.017	0.258	0.001	0.266	−0.103	0.134
Char12	−0.665	0.203	0.051	−0.142	0.184	0.160	−0.070	−0.054	0.123	0.075	0.067
Char13	0.041	0.044	−0.012	0.329	0.144	0.295	0.292	0.550	−0.149	−0.118	0.157
Char14	−0.090	0.556	0.276	0.150	−0.030	0.021	0.217	0.110	0.031	0.194	−0.491
Char15	−0.482	0.498	−0.246	0.238	0.108	0.215	0.079	−0.146	0.181	0.044	0.048
Char16	0.121	−0.035	−0.216	−0.111	0.095	0.151	−0.066	−0.016	0.086	0.665	0.302
Char17	−0.689	0.107	0.127	0.063	0.059	−0.066	0.481	−0.024	−0.134	0.051	0.005
Char18	0.258	−0.118	0.305	0.205	−0.421	0.195	0.360	−0.102	0.186	0.179	−0.096
Char19	0.137	0.383	0.520	−0.286	0.013	−0.019	−0.247	−0.139	−0.026	0.137	0.375
Char20	0.488	0.150	0.321	0.163	0.125	−0.499	0.002	0.133	0.120	−0.042	−0.160
Char21	0.039	0.085	−0.032	−0.617	0.064	0.146	0.297	−0.136	−0.321	−0.079	−0.016
Char22	0.298	0.578	0.305	−0.089	−0.036	−0.225	0.038	0.149	0.164	0.101	0.055
Char23	−0.106	0.524	−0.355	−0.458	0.016	0.140	0.078	0.224	−0.025	−0.047	−0.234
Char24	−0.109	0.017	−0.091	0.543	−0.335	−0.209	−0.191	0.167	−0.036	0.046	0.162
Char25	0.160	0.605	−0.427	0.043	0.016	0.052	−0.149	−0.016	−0.182	0.187	−0.040
Char26	−0.247	0.749	−0.162	0.056	0.008	−0.224	−0.055	0.062	−0.194	0.007	0.063
Char27	−0.201	0.385	0.117	0.341	−0.086	−0.071	−0.135	−0.383	−0.401	−0.088	0.234
Char28	−0.430	−0.142	0.416	−0.273	0.098	−0.103	−0.226	−0.058	0.122	0.117	−0.037
Char29	−0.078	0.482	0.256	−0.036	−0.061	0.026	−0.390	0.017	−0.054	−0.142	−0.194
Char30	0.518	0.099	0.150	0.202	0.188	0.277	−0.122	0.173	−0.057	−0.180	0.206
Char31	−0.068	−0.069	0.255	0.154	0.120	0.644	−0.319	−0.119	−0.036	−0.207	−0.155

**Table 3:** Comprehensive score and ranking of 183 *Setaria italica* accessions.

Variety Num	Score	Rank	Variety Num	Score	Rank	Variety Num	Score	Rank
144	0.0044019	1	47	0.0051848	62	75	0.0057543	123
164	0.0044072	2	18	0.0051848	63	80	0.0057656	124
169	0.0044475	3	58	0.0052139	64	9	0.0057692	125
163	0.0044789	4	160	0.0052141	65	180	0.0057746	126
178	0.0044814	5	109	0.0052163	66	28	0.0057757	127
136	0.0045208	6	147	0.0052227	67	123	0.0057808	128
23	0.0045217	7	82	0.0052237	68	12	0.0057846	129
24	0.0045440	8	63	0.0052340	69	97	0.0057949	130
130	0.0045671	9	79	0.0052358	70	99	0.0058321	131
161	0.0045847	10	64	0.0052430	71	153	0.0058577	132
2	0.0046025	11	33	0.0052437	72	13	0.0058617	133
137	0.0046308	12	166	0.0052499	73	110	0.0058849	134
168	0.0046707	13	10	0.0052500	74	98	0.0058937	135
129	0.0046859	14	31	0.0052550	75	172	0.0058989	136
73	0.0046881	15	158	0.0052550	76	39	0.0059003	137
106	0.0046898	16	142	0.0052580	77	182	0.0059397	138
4	0.0047016	17	135	0.0052580	78	72	0.0059493	139
133	0.0047340	18	60	0.0052635	79	59	0.0059500	140
175	0.0047354	19	19	0.0052735	80	29	0.0059534	141
167	0.0047465	20	132	0.0052810	81	68	0.0059650	142
138	0.0047536	21	173	0.0052846	82	35	0.0059703	143
113	0.0047543	22	8	0.0052868	83	49	0.0059762	144
5	0.0047684	23	126	0.0052929	84	124	0.0059786	145
177	0.0047778	24	52	0.0052994	85	89	0.0059938	146
1	0.0047928	25	17	0.0053017	86	102	0.0060098	147
131	0.0047944	26	65	0.0053155	87	122	0.0060135	148
61	0.0048162	27	88	0.0053402	88	85	0.0060177	149
174	0.0048341	28	127	0.0053587	89	30	0.0060186	150
171	0.0048344	29	118	0.0053686	90	48	0.0060246	151
159	0.0048400	30	146	0.0054040	91	93	0.0060310	152
162	0.0048416	31	62	0.0054062	92	108	0.0060672	153
145	0.0048555	32	96	0.0054110	93	46	0.0060870	154
128	0.0048679	33	56	0.0054122	94	40	0.0060899	155
157	0.0048728	34	7	0.0054256	95	91	0.0060950	156
22	0.0048753	35	16	0.0054256	96	27	0.0061222	157
134	0.0048994	36	57	0.0054285	97	151	0.0061299	158
179	0.0049497	37	120	0.0054855	98	37	0.0061510	159
156	0.0049500	38	84	0.0055166	99	104	0.0061545	160
77	0.0049523	39	155	0.0055502	100	140	0.0061572	161
11	0.0049550	40	141	0.0055515	101	181	0.0061625	162
6	0.0049585	41	76	0.0055526	102	36	0.0061794	163
15	0.0049806	42	41	0.0055599	103	38	0.0061982	164
71	0.0050105	43	83	0.0055611	104	107	0.0062144	165
66	0.0050126	44	183	0.0055832	105	149	0.0062277	166
165	0.0050212	45	152	0.0055913	106	44	0.0062539	167
114	0.0050214	46	125	0.0056023	107	90	0.0062543	168
143	0.0050331	47	139	0.0056143	108	34	0.0063022	169
170	0.0050404	48	50	0.0056196	109	43	0.0063163	170
95	0.0050498	49	55	0.0056255	110	53	0.0063217	171
21	0.0050560	50	74	0.0056296	111	150	0.0063330	172
25	0.0050603	51	154	0.0056363	112	111	0.0063364	173
20	0.0050791	52	69	0.0056388	113	103	0.0063750	174
78	0.0050879	53	119	0.0056447	114	100	0.0064080	175
87	0.0050928	54	81	0.0056666	115	92	0.0064706	176
14	0.0050977	55	51	0.0056849	116	94	0.0065087	177
3	0.0050979	56	54	0.0056977	117	45	0.0065936	178
112	0.0051050	57	148	0.0057056	118	32	0.0066193	179
121	0.0051128	58	115	0.0057134	119	42	0.0066241	180
67	0.0051468	59	26	0.0057135	120	86	0.0067931	181
116	0.0051541	60	70	0.0057182	121	101	0.0068839	182
176	0.0051605	61	117	0.0057442	122	105	0.0071148	183

**Table 4:** The information on the wheat DUS testing characteristics used in this study.

Characteristics	Character Code	Type of Expression	Method of Observation	States and Code of Expression
First leaf: shape of tip	char1	PQ	VG	pointed (1); pointed to rounded (2); rounded (3)
Seedling: leaf color	char2	PQ	VG	yellow-green (1); green (2); light purple (3); purple (4)
Seedling: leaf sheath color	char3	PQ	VG	green (1); light purple (2); medium purple (3)
Seeding: growth habit	char4	PQ	VG	upright (1); semi-upright (2); spreading (3); drooping (4)
Seedling: anthocyanin shows color in leaf midrib	char5	QN	VG	absent or weak (1); medium (2); strong (3)
Time of heading	char6	QN	MG	very early (1); early (3); medium (5); late (7); very late (9)
Plant: growth habit	char7	PQ	VG	upright (1); semi-upright (2); spreading (3); drooping (4)
Panicle: length of bristles	char8	QN	VG	short (3); medium (5); long (7)
Panicle: bristles color	char9	PQ	VG	green (1); yellow (2); purple (3)
Anther: color	char10	PQ	VG	white (1); yellow (2); brown (3)
Flag leaf: length of blade	char11	QN	MS/MG	short (1); medium (3); long (5)
Flag leaf: width of blade	char12	QN	MS/MG	narrow (1); medium (3); broad (5)
Panicle: color of glume	char13	PQ	VG	yellow-green (1); green (2); red (3); light purple (4); medium purple (5)
Stem: length	char14	QN	MS/MG	very short (1); short (3); medium (5); long (7); very long (9)
Stem: diameter	char15	QN	MS/MG	narrow (3); medium (5); broad (7)
Plant: color	char16	PQ	VG	yellow (1); green (2); light purple (3); medium purple (4)
Plant: number of elongated internodes	char17	QN	MG	few (1); medium (3); many (5)
Plant: number of culms per panicle	char18	QN	MS	few (1); medium (3); many (5)
Panicle neck: attitude	char19	PQ	VG	straight (1); medium curve (2); strong curve (3); claw (4)
Panicle neck: length	char20	QN	MS	short (3); medium (5); long (7)
Panicle: type	char21	PQ	VG	conical (1); spindle (2); cylindrical (3); club (4); duck mouth (5); cat foot (6); branched (7)
Panicle: length	char22	QN	MG	very short (1); short (3); medium (5); long (7); very long (9)
Panicle: diameter	char23	QN	MS	narrow (3); medium (5); broad (7)
Panicle: density	char24	QN	VG	lax (1); lax to medium (2); medium (3); medium to dense (4); dense (5)
Panicle: single-grain number	char25	QN	MG	very few (1); few (3); medium (5); many (7); very many (9)
Panicle: single panicle weight	char26	QN	MS	very low (1); low (3); medium (5); high (7); very high (9)
Panicle: grain yield per panicle	char27	QN	MS	low (1); medium (2); high (3)
1000 grain weight	char28	QN	MG	low (1); medium (2); high (3)
Grain: shape	char29	PQ	VG	narrow ovate (1); medium ovate (2); circular (3)
Grain: color	char30	PQ	VG	white (1); yellow (2); red (3); brown (4); gray (5); black (6)
Dehusked grain: color (not polished)	char31	PQ	VG	white (1); gray-green (2); light yellow (3); medium yellow (4); gray (5)
Endosperm: type	char32	QL	VG	waxy (1); non-waxy (2)

## Data Availability

The original contributions presented in this study are included in this article; further inquiries can be directed to the corresponding authors.
